# Alternative method to treat oroantral communication and fistula with autogenous bone graft and platelet rich firbin

**DOI:** 10.4317/medoral.21037

**Published:** 2016-07-31

**Authors:** Michał Kapustecki, Iwona Niedzielska, Halina Borgiel-Marek, Bartosz Różanowski

**Affiliations:** 1 Department of Cranio-Maxillofacial Surgery and Dental Surgery Silesian Medical University in Katowice, Poland; 2 Professor Medicine. Department of Cranio-Maxillofacial Surgery and Dental Surgery Silesian Medical University in Katowice, Poland; 3 DSc, PhD. Department of Cranio-Maxillofacial Surgery and Dental Surgery Silesian Medical University in Katowice, Poland; 4 DSc, PhD. Department of Cytology and Genetics, Institute of Biology, Pedagogical University, Kraków, Poland

## Abstract

**Background:**

Removing a tooth from the jaw results in the occurrence of oroantral communication in beneficial anatomic conditions or in the case of a iatrogenic effect. Popularized treatments of the oroantral communication have numerous faults. Large bone defect eliminates the chance to introduce an implant. Purpose of this work was assessment of the usefulness of autogenous bone graft and PRF in normal bone regeneration in the site of oroantral communication.

**Material and Methods:**

Bone regeneration in the site of oroantral communication was assessed in 20 patients. Bone defects were supplemented autogenous bone graft from mental protuberance in 14 cases and from oblique line in 6 cases. The graft was covered with a PRF membrane.

**Results:**

In the study group in all cases closure of the oroantral communication was observed. The average width of the alveolar was 13 mm and the average height was 12.5 mm. In 3 patients an average increase of alveolar height of 1.5 mm was observed.

**Conclusions:**

This method may be the best option to prepare alveolar for new implant and prosthetic solutions.

**Key words:**Oroantral communication, oroantral fistula, autogenous bone graft, bone regeneration, platelet rich fibrin.

## Introduction

Nowadays a lot of space is devoted to augmentation procedures regarding various types of surgical treatments, such as cystectomy, resection, extraction, pre-implantation treatments. Single-stage closure of an oroantral fistula and an increase in vertical and/or horizontal dimensions of the maxillary alveolar also deserve attention.

Traditional methods to close oroantral communication, which occur after removing a tooth from the jaw, performed directly or as a stage of surgical treatment of chronic maxillary sinusitis with plastic surgery of oroantral fistula involve the use of patches formed from soft tissue and their tight suturing. This procedure results in shallowing of vestibule and abnormal bone remodeling in the site of the oroantral defect which makes future prosthetic or implant and prosthetic treatment difficult or impossible. Thanks to modern reconstructive surgery which is developing very dynamically, we can consider reconstructing lost bone tissue in the site of the oroantral communication.

An oroantral communication is a complication which occurs most frequently during the extraction of the first and second molars, less frequently the third (1-6). The relation of oral cavity with maxillary sinus constitutes a gate for the mucosal infection in maxillary sinus. Presence of the defect is diagnosed during a clinical examination, performing a Valsalva maneuver, mechanical and water testing. Later symptoms also include leakage of purulent contents from the alveolus, epistaxis, speech and breathing disorders. Additional tests include radiographs (pantomographic, sinus PA) and computed tomography.

According to different authors, the frequency of such complications is between 0.5 and 13 per cent and it depends on numerous factors, such as anatomical structure of the maxillary sinus, occurrence of large periapical changes, tumors, cysts, mucous membrane inflammation in the maxillary sinus (Schneiderian membrane), a patient’s age and the tooth extraction method (2-5,7).

Immediate plastic surgery of the oroantral fistula is effective in 95 per cent, while a postponed surgery only in 67 per cent (4). In the light of recent studies and opinions, an oroantral fistula should be closed in 24 hours. After this period, in approximately half of the patients intensified inflammatory changes make it impossible to effectively conduct the treatment (4,8). In such cases a procedure of choice is a surgery of maxillary sinus together with plastic surgery of the oroantral communication.

It can be concluded from clinical observations that closing the defect even after a few days can be successful. It is however subject to the presence of mucous membrane unchanged by inflammation and lack of foreign bodies in the form of for example a pushed root or endodontic material. For this purpose endoscopic revision, X-rays and sinus washing are conducted (1).

It occurs sometimes that the oroantral communication especially of a small size (up to 5 mm) undergoes spontaneous healing provided that the sinus and the clot filling the alveolus are clean. However, this is not a method recommended by clinicians (2,4,5).

Considering current opinions, each oroantral communication should be treated surgically after previous diagnostics which excludes the presence of a foreign body and/or inflammatory changes of mucous membrane. An undiagnosed oroantral communication or treatment of an oroantral communication followed by complications results in chronic maxillary sinusitis. Its treatment involves removal of the oroantral fistula, surgery of the maxillary sinus and closure of the oroantral defect (1,2,5).

The clinical practice most frequently involves the monolayer technique of closing an oroantral defect developed in 1939 by Wassmund, and modified in 1948 by Borusiewicz (1,2).

A disadvantage of this method is oral cavity vestibule shallowing and alveolar bone loss which limits the prosthetic substrate in this area.

An arterialized palatal flap, a flap moved from the cheek and a Zange flap operation technique or non-flap techniques using resorbable polyurethane sponges in the form of a cone are less frequently used methods of closing oroantral communication (5,8-14).

A significant fault of the above mentioned monolayer methods is the formation of a mucosal bridge without normal bone regeneration.

In the case of large communications or fistulas in patients after radiotherapy, the closure of which using classical methods did not succeed, a bilayer method using buccal fat pad maintaining vascular pedicle has found its application. In the absence of general contraindications autogenic, allogenic, xenogeneic and alloplastic materials (different types of gore-tex mesh, titanium mesh, metal plates and foils, Zanoderm - freeze-dried pork skin) can be used as a deep layer (11,15-17).

In especially difficult cases closure of the defect can be performed with the use of a tongue flap, periosteal flap and cartilage collected from nasal septum, temporal fascia or conducting an autotransplant of the third molar in place of the oroantral fistula (5).

An oroantral communication with large alveolar damage operated with the use of monolayer or bilayer method without using the graft material influences impaired bone remodeling (resorption), simultaneously disqualifying the patient from single-stage implant and prosthetic treatment.

To meet the contemporary requirements the repair method of an oroantral communication with single-stage alveolar augmentation with autogenous bone graft and PRF has found its use (17,18).

The purpose of this dissertation is to assess the usefulness of autogenous bone graft collected from the mandible in the area the oblique line and mental protuberance, and platelet rich fibrin in normal bone regeneration in oroantral communication.

## Material and Methods

The study included a group of 20 patients of the Oral and Maxillofacial Surgery Outpatient Clinic in Katowice who were diagnosed with oroantral communication. On the basis of clinical examinations and X-rays: Pantomographic, radially adjacent images, Waters X-ray and CBCT the studies included patients in whom the following conditions were diagnosed.

- Connection of the oral cavity and the maxillary sinus in the neighborhood of 2 teeth (up to 48 hours).

- An oroantral fistula in the neighborhood of 2 teeth (up to 2 weeks).

- Preserved bone lamellae: vestibular and palatal.

The following patients were excluded from the studies.

- Patients with a foreign body in the maxillary sinus.

- Patients with inflammation of the maxillary sinus in the medical history, confirmed on Waters X-ray or during water testing.

 - Systemic diseases (diabetes, nephropathy, coagulation disorders, autoimmune diseases, cancers).

In each case quality and quantity bacteriological examinations of the material collected from the maxillary sinus and antibiogram were conducted.

The study was approved by the Ethics Committee of the Silesian Medical University in Katowice and is in compliance with the Helsinki Declaration (KNW/0022/KB1/97/II/10/11). All patients were informed of the study procedure and gave their signed consent to take part; the study was designed to ensure that all current ethical and legal requirements would be met.

In order to prepare a PRF membrane in each patient 3-5 venous blood test-tubes with a volume of 9 ml were collected; they were centrifuged for 12 minutes at a speed of 2700 rpm. A rich platelet fibrin clot was isolated from the middle part of the test-tube between Red Base Corpuscles at the bottom and Platelet-Poor Plasma at the top, and then it was filtered from the plasma in the form of a thin membrane.

**Figure 1:**
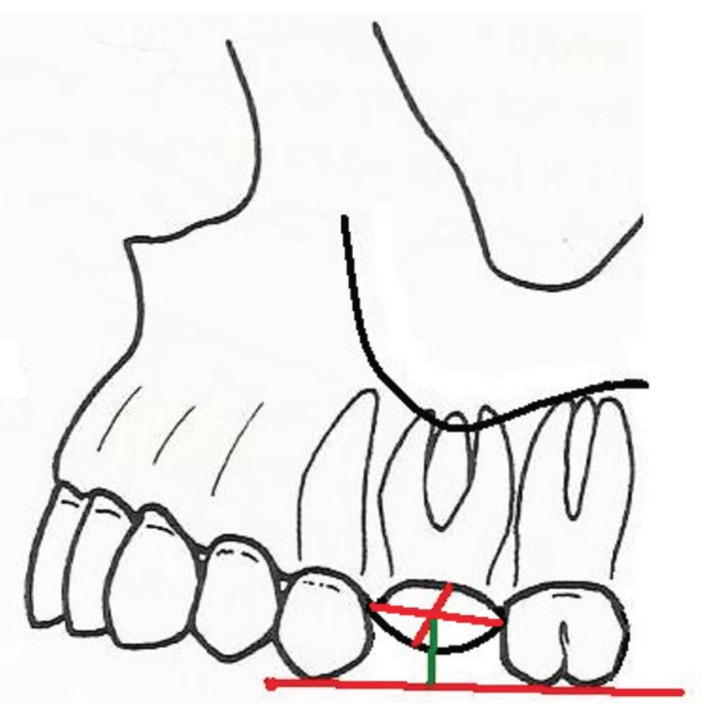
H m (green) - distance from IC line to the measurement point inside the alveolus (the point of intersection of the line connecting the edges of the alveolus in both spatial directions - red) IC line (red) -cusps of teeth neighboring with alveolus.

**Figure 2:**
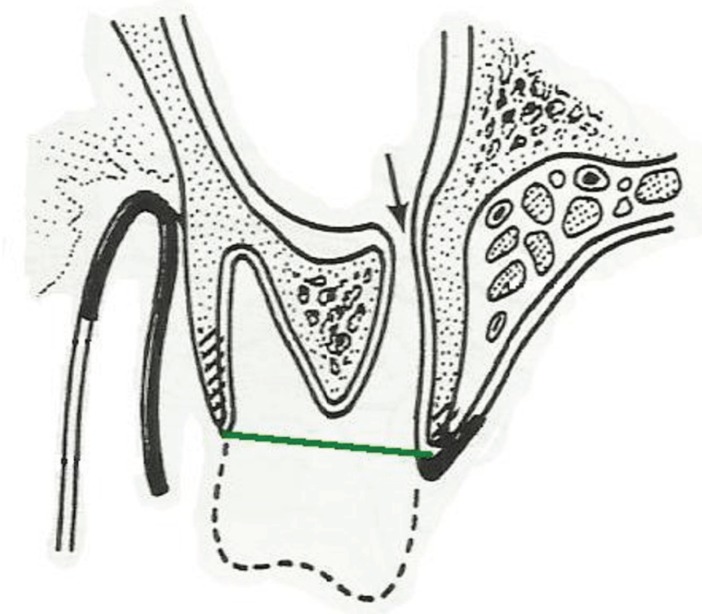
Measurement of the width of alveolar in the lateral projection of alveolus - green line (H v-p).

Closure of the oroantral communication was conducted under local anesthesia. The first stage involved the formation of a trapezoidal muco-periosteal flap in the oral cavity vestibule, as well as revision and cleansing of the post-extraction wound. Then, measurements of the alveolar width in the transversal projection of alveolus and measurements of the average height of alveolar bone lamina from the side of the oral cavity vestibule and from the side of the palate were conducted intraoperatively. (Figs. 1,2).

**Figure 3:**
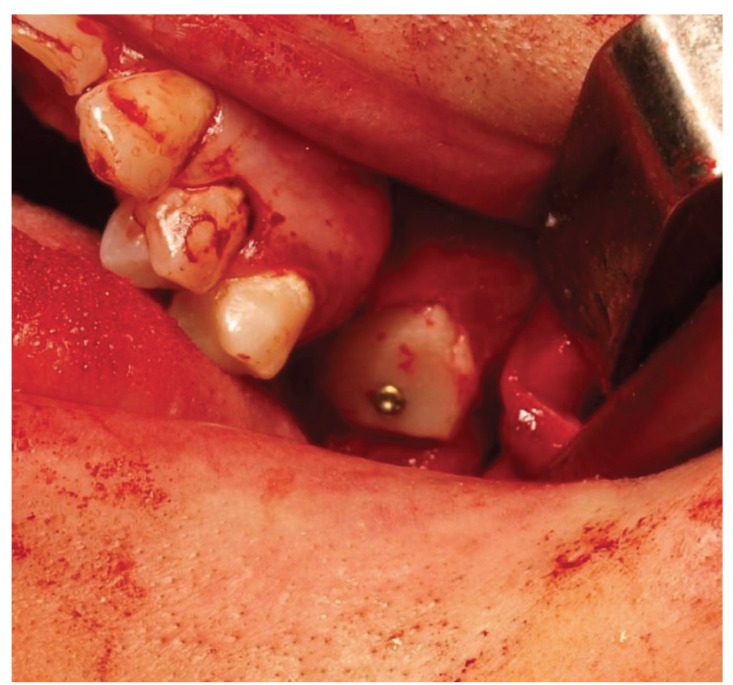
Bone graft fixed bicortical screw.

The next stage depending on the cavity diameter involved collection of monocortical bone blocks from mental protuberance (14 patients) or mandible oblique line (6 patients) with the use of trephine or a piezoelectric knife. The bone blocks were shaped in a way which made it possible to wedge them in the cavity and tightly close the defect. The graft was stabilized using a bicortical screw or a titanium mini-plate of the MEDARTIS 1.5 system, levelling its mobility. (Fig. 3). Sharp bone edges were smoothed. The graft and surrounding bone were covered with a PRF membrane. The whole was tightly sutured without tension the vestibule flap.

The first measurement was conducted intraoperatively and the next one 3-6 months after during the removal of the stabilizing element.

The healing process of soft tissue in the postoperative area, temporary sensory disturbances, unveiling of the aggregating element, loss of graft stability, occurrence of a secondary oroantral fistula were also assessed. The follow-up was conducted 2 weeks after surgery and then again 3 and 6 months after surgery.

## Results

In 14 patients bone block was collected from mental protuberance and from oblique line in 6 patients. The reason for the oroantral communication was always the removal of the first maxillary molar tooth. In the study group in all patients closure of the oroantral communication took place. The average width of the alveolar in the area of the removed tooth before introducing the graft was 13.5 mm, while the average distance between the measurement points determining the value of a vertical alveolar atrophy was 13 mm.

Clinical assessment conducted 2 weeks after the surgery indicated complete healing of the operated area in 18 patients; in 2 patients unveiling of the graft stabilizing screw was observed. Due to its small surface the screw was left in place, and enhanced oral hygiene was recommended. No inflammation, exposure or graft stabilization loss was observed. In the follow-up conducted after 3 months in 3 patients mobility of aggregating elements was observed, including 1 patient with inflammation related to it. The stabilizing elements were removed. In the remaining cases the stabilizing elements were removed after 6 months. During the removal operation bone remodeling was assessed and measurements were conducted. The average width of alveolar was 13 mm, and the average distance between the measurement points determining the value of a vertical alveolar atrophy was 12.5 mm. In 3 patients the value was less than 11 mm. (Tables  Table 1, Table 2)

**Table 1:**
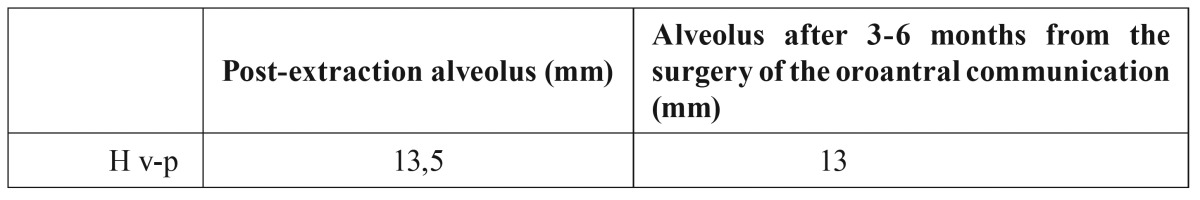
Mean measurement alveolar width values in a group of 20 patients.

**Table 2:**
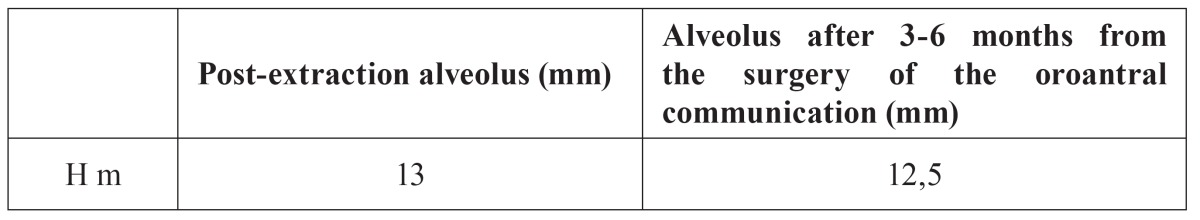
Mean measurement alveolar height values in a group of 20 patients.

Radiological evaluation conducted after 6 months from the surgery indicated the presence of a non-resorbed bone graft and no inflammation within the maxillary sinus.

## Discussion

Considering current study results and opinions, autogenous bone grafts are a golden standard of treating bone loss and defects. In defined clinical cases they are displaced by allogenic, alloplastic and xenogeneic implants. Both from biological and immunological, as well as from legal and ethical points of view and due to the lack of risk of cross-contamination, it is recommended to use autogenous bone (17). Biological properties of autogenous bone grafts are related to osteocunductivity, osteoinduction and osteogenesis processes. Most of all, it is recommended to use autogenous bone grafts in pre-implantation treatments in order to increase vertical and horizontal dimensions of the alveolar and in reconstruction of craniofacial bone loss after extensive cancer treatments, cyst removal and in orthognathic defect treatment.

A site for large graft collection is most frequently a hip bone, a rib, scapula, tibia, fibula or parietal. In the majority of dental procedures bone is collected intraorally from mandible oblique line, mental protuberance, postmolar area, anterior maxillary sinus wall, zygomaticoalveolar crest or mandible casp. The faults related to the collection of autogenous graft include: creation of another operating field, bone weakening in the donor site, extension of the treatment duration (5,17,18).

PRF (platelet-rich fibrin) is a product of centrifuged blood. Biochemical analysis of PRF composition indicates that this biomaterial consists of accumulated cytokines, glycan chains and glycoprotein structures inside the slowly polymerized fibrin network. These biochemical components are well-known as factors acting synergistically in the healing process. They include: Platelet-Derived Growth Factor (PDGF), Transforming Growth Factor beta (TGFβ), Insuline Growth Factor (IGF). In a PRF clot there are many cytokines included: inflammation cytokines, such as IL-6, IL-1β, IL-1, TNF-α, and healing cytokines, such as IL-4 and VEGF. These components are the reason why PRF has anti-inflammatory properties, as well as it accelerates angiogenesis and creation of fibroblasts and osteoblasts, the consequence of which is the enhanced healing process. Obtaining PRF is only related to a minimally invasive procedure of venous blood collection (19,20).

The technique to close the oroantral communication using autogenous bone graft and a PRF membrane can constitute an interesting alternative to traditional single- and bilayer procedures to close oroantral communication. It provides numerous benefits related to prosthetic and implant treatment. It allows the alveolar shape to be maintained and even to increase its vertical dimension. The average decrease of the H v-p dimension value for the alveolar without a graft is 4.4 mm, which constitutes a significant difference in comparison to the one observed in own studies and studies conducted by different authors (21). A well-profiled graft acts as a form of plug, securing from pressure changes in paranasal sinuses. The PRF membrane covers the graft, while the above mentioned components contained in it have positive impact on its integration. The majority of authors of publications regarding this issue use resorbable collagen membranes or non-resorbable membranes separating the graft from the maxillary sinus (18,22-24). Therefore, this method may turn out to be another way for single-stage closure of a oroantral communication and alveolar augmentation. Introducing this technique to be used commonly requires further prospective clinical trials.
